# Absence of spontaneous disease and comparative prion susceptibility of transgenic mice expressing mutant human prion proteins

**DOI:** 10.1099/vir.0.007930-0

**Published:** 2009-03

**Authors:** Emmanuel A. Asante, Ian Gowland, Andrew Grimshaw, Jacqueline M. Linehan, Michelle Smidak, Richard Houghton, Olufunmilayo Osiguwa, Andrew Tomlinson, Susan Joiner, Sebastian Brandner, Jonathan D. F. Wadsworth, John Collinge

**Affiliations:** MRC Prion Unit and Department of Neurodegenerative Disease, UCL Institute of Neurology, National Hospital for Neurology and Neurosurgery, Queen Square, London WC1N 3BG, UK

## Abstract

Approximately 15 % of human prion disease is associated with autosomal-dominant pathogenic mutations in the prion protein (PrP) gene. Previous attempts to model these diseases in mice have expressed human PrP mutations in murine PrP, but this may have different structural consequences. Here, we describe transgenic mice expressing human PrP with P102L or E200K mutations and methionine (M) at the polymorphic residue 129. Although no spontaneous disease developed in aged animals, these mice were readily susceptible to prion infection from patients with the homotypic pathogenic mutation. However, while variant Creutzfeldt–Jakob disease (CJD) prions transmitted infection efficiently to both lines of mice, markedly different susceptibilities to classical (sporadic and iatrogenic) CJD prions were observed. Prions from E200K and classical CJD M129 homozygous patients, transmitted disease with equivalent efficiencies and short incubation periods in human PrP 200K, 129M transgenic mice. However, mismatch at residue 129 between inoculum and host dramatically increased the incubation period. In human PrP 102L, 129M transgenic mice, short disease incubation periods were only observed with transmissions of prions from P102L patients, whereas classical CJD prions showed prolonged and variable incubation periods irrespective of the codon 129 genotype. Analysis of disease-related PrP (PrP^Sc^) showed marked alteration in the PrP^Sc^ glycoform ratio propagated after transmission of classical CJD prions, consistent with the PrP point mutations directly influencing PrP^Sc^ assembly. These data indicate that P102L or E200K mutations of human PrP have differing effects on prion propagation that depend upon prion strain type and can be significantly influenced by mismatch at the polymorphic residue 129.

## INTRODUCTION

Inherited prion diseases (IPDs) are fatal neurodegenerative disorders caused by autosomal-dominant mutations in the human PrP gene (*PRNP*), and constitute about 15 % of all human prion disease (Collinge, 2001, 2005; Kovacs *et al.*, 2002; Wadsworth *et al.*, 2003; Mead, 2006). Over 30 mutations have been identified, but the precise biochemical mechanisms that lead to disease remain unknown.

According to the ‘protein-only’ hypothesis (Griffith, 1967), an abnormal isoform of host-encoded cellular prion protein (PrP^C^) is the principal, and possibly the sole, constituent of the transmissible agent or prion (Prusiner, 1982; Collinge & Clarke, 2007). This hypothesis proposes that the central pathogenic process is the conversion of PrP^C^ to a disease-related isoform, PrP^Sc^, through a conformational change occurring either spontaneously or during interaction with exogenous PrP^Sc^. PrP^Sc^ consists of aggregated misfolded PrP and distinct prion strains are thought to be composed of different polymeric forms of PrP (for recent review see Collinge & Clarke, 2007). Importantly, IPDs may also be experimentally transmissible and it is thought that pathogenic mutations in *PRNP* predispose mutant PrP^C^ to convert spontaneously to a pathogenic isoform (Collinge & Palmer, 1992; Collinge, 2005; Collinge & Clarke, 2007). While patients with IPD have traditionally been classified by the clinicopathological syndromes of Gerstmann–Sträussler–Scheinker disease (GSS), Creutzfeldt–Jakob disease (CJD) or fatal familial insomnia (FFI), the advent of molecular genetic diagnosis led to the recognition of considerable phenotypic heterogeneity even within families with the same *PRNP* mutation (Collinge *et al.*, 1989, 1990, 1992; Mallucci *et al.*, 1999; Kovacs *et al.*, 2002; Wadsworth *et al.*, 2006) and subclassification of IPD by pathogenic mutation was proposed (Collinge *et al.*, 1992; Collinge & Prusiner, 1992).

The mutation at codon 200 of *PRNP*, which results in a glutamic acid substitution by lysine (E200K) in PrP, is one of the most prevalent, being responsible for the high incidence of CJD amongst Libyan Jews and in areas of Slovakia and Chile, and is recognized in many other countries (Goldfarb *et al.*, 1990, 1991; Hsiao *et al.*, 1991; Brown *et al.*, 1992; Collinge *et al.*, 1993; Kovacs *et al.*, 2002). Families with the E200K mutation demonstrate varied clinical symptoms including uncommon features such as fatal insomnia, pruritus and demyelinating peripheral neuropathy or protracted dementia without other distinguishing characteristics (Chapman *et al.*, 1993). Indeed, this form of IPD can present clinically and pathologically like classic sporadic CJD (Chapman *et al.*, 1993), and although *PRNP* E200K homozygotes do not seem to differ in clinical features from heterozygotes, a statistically significant younger age at disease onset was found for homozygotes (Simon *et al.*, 2000). Penetrance for the E200K mutation is age dependent and approaches 100 % by 85 years of age (Chapman *et al.*, 1993).

A proline to leucine substitution at codon 102 (P102L) of human PrP is the most common mutation associated with the GSS phenotype and was first reported in 1989 (Hsiao *et al.*, 1989). Many other families have now been documented worldwide (Kovacs *et al.*, 2002), including the original Austrian family reported by Gerstmann, Sträussler and Scheinker in 1936 (Kretzschmar *et al.*, 1991; Hainfellner *et al.*, 1995). Progressive ataxia is the dominant clinical feature, with dementia and pyramidal features occurring later in a disease course typically much longer than that of classical CJD. However, marked variability at both the clinical and neuropathological levels is apparent, with some patients developing a classical CJD-like phenotype with early and rapidly progressive dementia (Hainfellner *et al.*, 1995; Barbanti *et al.*, 1996; Majtenyi *et al.*, 2000; Wadsworth *et al.*, 2006; Kretzschmar *et al.*, 1992; Webb *et al.*, 2008). A recent study indicates that differential recruitment of wild-type PrP into PrP^Sc^ may contribute to phenotypic variability in atypical cases (Wadsworth *et al.*, 2006).

Polymorphism at residue 129 of human PrP [where either methionine (M) or valine (V) can be encoded] not only affects susceptibility to sporadic and acquired human prion diseases (Palmer *et al.*, 1991; Collinge *et al.*, 1991; Lee *et al.*, 2001; Mead *et al.*, 2003), but can affect the age of onset and also modify the phenotypes of IPDs (Baker *et al.*, 1991; Poulter *et al.*, 1992; Goldfarb *et al.*, 1992; Mead *et al.*, 2006).

Early attempts to transmit IPDs in non-human primates (Brown *et al.*, 1994) and wild-type mice (Tateishi *et al.*, 1996) resulted in poor transmission rates, resulting in the important issue of whether or not all IPDs are experimentally transmissible being unresolved (Collinge, 1997).

Transgenic mice expressing high levels of mouse PrP 101L (equivalent to 102L in human PrP) spontaneously developed neurological dysfunction at 166 days of age (Hsiao *et al.*, 1990). PrP^Sc^ levels were low or undetectable, and brain extracts from affected mice did not transmit CNS degeneration to wild-type mice, but transmission to hamsters and Tg(GSSPrP)196 mice, expressing lower levels of the same mutant transgene product, was reported (Hsiao *et al.*, 1994; Telling *et al.*, 1996a). These Tg(GSSPrP)196 mice have subsequently been reported to develop spontaneous disease at advanced age (Tremblay *et al.*, 2004; Nazor *et al.*, 2005). It therefore remains debateable as to whether prions had been generated in these transgenic mice or this simply represents acceleration of a spontaneous neurodegenerative disease already poised to occur in these mice (Nazor *et al.*, 2005). Others generated transgenic mice expressing endogenous levels of mouse PrP 101L by the gene knock-in approach (Manson *et al.*, 1999). These mice did not develop spontaneous neurodegeneration but were reported to show greater susceptibility to human P102L prions than wild-type mice (Barron *et al.*, 2001).

However, we consider it essential to study this and other human pathogenic mutations in human PrP, rather than in mouse PrP where the mutation may have different structural consequences. With respect to such models it is important to demonstrate that human PrP is functionally active and can participate in prion propagation and pathogenesis in mouse cells. Human PrP can rescue a PrP null phenotype in mice (Whittington *et al.*, 1995), confirming it is functionally active and human prions can replicate in transgenic mice expressing only human PrP, which develop spongiform neurodegeneration (Collinge *et al.*, 1995).

Importantly, there are examples of IPD where the amino acid change thought to be pathogenic is found as a normal variant in other mammalian species (Butefisch *et al.*, 2000; Lysek *et al.*, 2004; Colucci *et al.*, 2006). There is also direct experimental evidence that a human *PRNP* mutation on a mouse background would not necessarily have the same structural consequences in the expressed protein. The introduction of a tryptophan residue at amino acid position 175 in place of the native phenylalanine has been successfully used as an optical probe for studying the folding dynamics of the recombinantly expressed mouse PrP (Wildegger *et al.*, 1999). The introduction of this probe had no measurable effect on the stability of the protein. However, in stark contrast, when we introduced the same mutation into the human *PRNP* gene, the resultant recombinant PrP was unable to fold into the native conformation (T. Hart, G. J. Jackson, A. R. Clarke & J. Collinge, unpublished data). The profoundly dissimilar consequences of the same mutation in mouse and human PrP questions the whole approach of modelling human pathogenic mutations on non-homologous PrP sequences from other species. In particular, the destabilizing effects measured in a mouse protein cannot be assumed to be equivalent in the human protein. The present study differs from all previous reports because we have investigated the biological properties of naturally occurring mutations in human PrP itself expressed in transgenic mice.

We have now generated two transgenic mouse lines that are both homozygous for the human PrP^102L,129M^ expressing transgene on a homozygous mouse PrP gene (*Prnp*) null background (HuPrP^102L,129M+/+^ *Prnp*^o/o^). Similarly, we have generated two further transgenic lines that are both homozygous for human PrP^200K,129M^ transgenes, again on a *Prnp*^o/o^ background (HuPrP^200K,129M+/+^ *Prnp*^o/o^). Here, we report the relative susceptibilities of these transgenic mice to classical (sporadic and iatrogenic) CJD prions, homotypic IPD isolates and variant (v) CJD prions.

## METHODS

### Transgene construction.

Briefly, the 759 bp human PrP ORF was amplified by PCR with *Pfu* polymerase from human genomic DNA with appropriate mutations, using forward primer 5′-GTCGACCAGTCATTATGGCGAACCTT-3′ and reverse primer 5′-CTCGAGAAGACCTTCCTCATCCCACT-3′. Restriction sites *Sal*I and *Xho*I (underlined) were introduced in the forward and reverse primers, respectively, for use in subsequent cloning steps. The blunt-ended PCR fragments generated by *Pfu* polymerase were subcloned into *Sma*I-digested pSP72 vector and sequenced to ensure that no spurious alterations have been introduced by the PCR other than the expected existing point mutations. The mutant human PrP ORFs with the appropriate point mutation confirmed were then isolated by using *Sal*I and *Xho*I. Subsequent subcloning into the cosmid vector SHaCosTt (Scott *et al.*, 1989) and preparation of high quality DNA of the insert was as described previously (Asante *et al.*, 2002).

### Microinjection.

The purified *PRNP* transgenes for P102L and E200K both with M at polymorphic codon 129 were microinjected (Hogan *et al.*, 1994) into single cell eggs of a strain of mice (FVB×SV129×C57) in which the murine PrP gene has been ablated. This was achieved by back-crossing ZH1 *Prnp* knockout line (Bueler *et al.*, 1992) to FVB/N for five generations, breeding out *Prnp* and restoring homozygosity of the knockout allele. The injected eggs were cultured to the two-cell stage and then surgically transferred to F1 (CBA×C57BL/6) recipient females. Two homozygous lines were established for P102L designated Tg27 and Tg33 with mutant transgene expression levels of three and one and a half times, respectively, compared with pooled normal human brain levels. Similarly, two homozygous lines were established for E200K designated Tg23 and Tg49 with relative expression levels of three- and twofold, respectively.

### Genotyping.

Tail biopsies were taken from putative transgenics and screened by PCR using human PrP-specific primers (5′-GTGGCCAGATGGAGTGACCTGGGCCTC-3′ and 5′-GGCACTTCCCAGCATGTAGCCG-3′). Founders were confirmed by Southern blotting using a 900 bp 3′ UTR fragment as a radiolabelled probe. Lines were bred to homozygosity and 20 mice were set aside for long-term neurological observation.

### Transmission studies.

All procedures were carried out in a microbiological containment level III facility with strict adherence to safety protocols. A panel of inocula comprising four inherited P102L, three sporadic and three iatrogenic CJD cases were used for the 102L transgenic mice. For the 200K transgenic mice, two inherited E200K and one iatrogenic CJD inocula were used. Inocula were prepared from the brain of neuropathologically confirmed cases of sporadic and inherited CJD with consent from relatives and with the approval from the Institute of Neurology/National Hospital for Neurology and Neurosurgery Local Research Ethics Committee. Mice were anaesthetized with a mixture of halothane and O_2_, and intracerebrally inoculated into the right parietal lobe with 30 μl of a 1 % (w/v) brain homogenate prepared in PBS. All mice were thereafter examined daily for clinical signs of prion disease. Mice were killed if exhibiting any signs of distress or once a diagnosis of prion disease was established.

### Neuropathology and immunohistochemistry.

Neuropathology and immunohistochemical analyses were done as described previously (Asante *et al.*, 2002, 2006) with the exception that abnormal PrP accumulation was examined using anti-PrP monoclonal antibody ICSM 18 (D-Gen Ltd) for P102L detection, and ICSM 35 (D-Gen Ltd) was used for E200K detection because the latter antibody does not recognize human PrP 102L (Wadsworth *et al.*, 2006). Appropriate controls were used throughout.

### Immunoblotting.

Brain homogenates (10 %, w/v) were prepared in Dulbecco's PBS lacking Ca^2+^ or Mg^2+^ ions (D-PBS) by serial passage through needles of decreasing diameter. Aliquots were analysed with or without proteinase K digestion (50 or 100 μg ml^−1^ final protease concentration, 1 h, 37 °C) by electrophoresis and immunoblotting as described previously (Wadsworth *et al.*, 2001, 2008; Hill *et al.*, 2003, 2006). Blots were probed with anti-PrP monoclonal antibody 3F4 (Kascsak *et al.*, 1987). For quantification and analysis of PrP glycoforms, blots were developed in chemifluorescent substrate (AttoPhos; Promega) and visualized on a Storm 840 phosphoimager (Molecular Dynamics) (Wadsworth *et al.*, 2001; Hill *et al.*, 2003, 2006). Quantification of PrP^Sc^ glycoforms was performed using ImageQuant software (Molecular Dynamics).

## RESULTS

### Transgenic mice expressing HuPrP 102L or 200K do not develop spontaneous disease

We generated two homozygous transgenic mouse lines designated Tg(HuPrP^102L,129M+/+^ *Prnp*^o/o^)-27 and Tg(HuPrP^102L,129M+/+^ *Prnp*^o/o^)-33 that expressed human PrP 102L,129M at three and one and a half times the human endogenous PrP^C^ levels, respectively (Table 1), but not mouse PrP.

In order to assess the possibility of spontaneous neurodegeneration, we monitored an ageing cohort of 20 uninoculated mice from each 102LL (homozygous for proline to leucine 102 mutation) transgenic line. Mice that died earlier than 400 days from intercurrent illnesses showed no evidence of prion-related neuropathology. Importantly, mice in the cohort survived to advanced age without clinical or neuropathological signs of prion disease or detectable PrP^Sc^, with the oldest mouse living to more than 865 days (mean survival shown in Table 1).

We also established two homozygous transgenic lines designated Tg(HuPrP^200K,129M+/+^ *Prnp*^o/o^)-23 and Tg(HuPrP^200K,129M+/+^ *Prnp*^o/o^)-49 that expressed human PrP 200K,129M at three and two times, respectively (Table 1), compared with endogenous PrP^C^ levels in a pooled normal human brain homogenate. We again set aside 20 mutant mice from both 200KK (homozygous for glutamic acid to lysine 200 mutation) lines and observed them long-term. Mice that died earlier than 400 days from intercurrent illnesses showed no evidence of prion-related neuropathology. Again, mice in the cohort survived to an advanced age without clinical or neuropathological signs of prion disease or detectable PrP^Sc^, with the oldest mouse living to more than 955 days (mean survival shown in Table 1). For both 102LL and 200KK mutant *PRNP* transgenic lines, Western blot analysis (data not shown) of samples from the brain of uninoculated mice showed equivalent proportions of di-, mono- and non-glycosylated PrP to that seen in Tg35 and Tg45 transgenic mice expressing wild-type human PrP 129M (Asante *et al.*, 2002). These data establish that the *PRNP* point mutations do not selectively destabilize a particular PrP glycoform. It is worth noting that Tg45 mice expressing human PrP 129M at four times wild-type levels do not develop spontaneous disease at a similar age (Asante *et al.*, 2002).

### Transgenic mice expressing HuPrP 102L are more susceptible to prions from patients with IPD (P102L) than to classical CJD prions

To assess the susceptibility of human PrP 102L-expressing transgenic mice to human prions, we inoculated groups of Tg27 and Tg33 mice intracerebrally with isolates from patients with classical CJD and IPD (P102L). Clinical disease with high attack rates and short incubation periods ranging from 185 to 191 days accompanied the transmission of four different IPD P102L cases to Tg27 transgenic mice (Table 2). In sharp contrast, challenge of Tg27 mice with four classical CJD isolates, all resulted in incomplete clinical attack rates and prolonged and highly variable incubation periods ranging from 342 to 717 days. However, with the exception of one inoculum, I026, where two mice were not affected, all sporadic and iatrogenic CJD-inoculated mice were scored as positive for prion infection by one or more of the following criteria: typical clinical signs, presence of PrP^Sc^ on Western blot analysis or abnormal PrP immunohistochemistry (Table 2).

A similar pattern of differential susceptibility to prions from patients with IPD P102L and classical CJD was also observed in Tg33 mice, which have lower expression of human PrP 102L (Table 3). However, compared with Tg27, the incubation periods for prions from patients with IPD P102L in Tg33 mice were longer by about 160 days and clinical attack rates were lower, but this difference is consistent with the lower transgene expression levels in this line of transgenic mice. Importantly, the apparent transmission barrier to classical CJD relative to IPD P102L prions was again clearly observed, with mean incubation periods for sporadic and iatrogenic CJD prions being about 200 days longer than for IPD P102L cases (Table 3).

Neuropathological examination was performed to investigate whether the differential susceptibility to prions from classical CJD and IPD P102L cases would be associated with the development of distinct patterns of neuropathology. However, there was no discernible neuropathological difference between Tg27 and Tg33 mice challenged with either classical CJD or inherited P102L prions (data not shown). Using Tg27 as a representative line, transmissions of both classical and IPD P102L prions were characterized by generalized synaptic PrP deposits in the cerebral cortex, basal ganglia, hippocampus, thalamus (Fig. 2d[Fig f2]) and to a lesser extent, in the brain stem and cerebellum (Fig. 2a, b[Fig f2]). The main difference between classical CJD- and IPD P102L-prion-inoculated transgenic mice was quantitative, in that mice inoculated with classical CJD prions had more intense staining than recipients of IPD P102L prions (Fig. 2a, b[Fig f2]). In mice with high levels of PrP deposition, PrP deposits were evident in the white matter. There was widespread spongiosis in almost all grey matter areas, with only the frontal lobes being spared. Multicentric plaques, a characteristic feature of GSS and associated with IPD P102L neuropathology was not seen in our models, with punctate PrP staining only being observed in the hippocampus (Fig. 2e[Fig f2]).

### Transgenic mice expressing HuPrP 200K show similar susceptibilities to IPD (E200K) and classical CJD prions

Groups of Tg23 and Tg49 transgenic mice were challenged with brain homogenates from two different human IPD E200K cases, one homozygous for *PRNP* 129M (designated E200K-129MM) and the other homozygous for *PRNP* 129V (designated E200K-129VV). In addition, we transmitted one case of iatrogenic CJD to both lines in order to investigate its interactions with human PrP 200K.

IPD E200K-129MM inoculum (I1091) transmitted clinical disease to 8/8 of Tg23 mice, with a short mean incubation period of 184±3 days (Table 4). In sharp contrast, the second IPD E200K inoculum (I093) (E200K-129VV) produced clinical disease in only 1/4 inoculated mice with a relatively prolonged incubation period of 437 days. There was however 100 % total infection rate because the three clinically asymptomatic mice, which died at 410, 518 and 538 days post-inoculation, had clear evidence of subclinical prion infection as determined either by PrP immunohistochemical or Western blot analysis (Table 4). The transmission properties of classical CJD in Tg23 mice were distinct from that observed in human PrP 102L-expressing transgenic mice. Here, iatrogenic CJD 129MM inoculum (I026) produced 100 % clinical attack rate and with relatively short mean incubation period of 184±7 days that was remarkably similar to the mean incubation period for IPD E200K-129MM inoculum I1091. These data suggest that, providing there is homology at residue 129 between the inoculum and the host PrP, the E200K mutation does not introduce a transmission barrier for classical CJD prions.

The prion transmission pattern for transgenic line Tg49 that had a lower level of expression of human PrP 200K, also followed a similar trend, being characterized by high clinical attack rates and almost identical incubation periods of 344 days for iatrogenic CJD inoculum I026, and 348 days for IPD E200K-129MM inoculum I1091 (Table 5). The transmission barrier associated with 129V was also evident in Tg49 mice, as challenge of this line with IPD E200K-129VV inoculum (I093) was also characterized by only 1/6 clinical attack rate and a prolonged incubation period of 552 days, about 200 days longer than that observed for iatrogenic CJD (129MM) in the same line (Table 5). The apparent low transmission efficiency of IPD E200K-129VV inoculum (I093) in both Tg23 and Tg49 mice was not due to low prion titre, because the same inoculum caused clinical disease in 11/11 Tg152 transgenic mice expressing human PrP 129V with a short incubation period of 187±9 days (data not shown). PBS-inoculated transgenic mice did not develop clinical disease, with most mice dying from intercurrent illnesses at advanced ages with no neuropathology.

Neuropathological examination of affected brains revealed synaptic type PrP deposits in the cortex and thalamic nuclei of iatrogenic CJD-inoculated Tg23 and Tg49 mice (data not shown), and this pattern of neuropathology closely resembled that produced in both transgenic lines by IPD E200K-129MM inoculum (I1091) (Fig. 2g, j[Fig f2]). Interestingly, the only discrete PrP plaques seen were associated with IPD E200K-129VV inoculum I093 and these were predominantly located in the cortex and corpus callosum (Fig. 2h, k[Fig f2]). There were comparatively more of these discrete PrP plaques in the Tg49 transgenic line with lower expression level (Fig. 2h, k[Fig f2]) than in the higher expressing Tg23 line (data not shown).

### PrP glycoform profiles in transgenic mice challenged with classical CJD and IPD prions

**Fig. 1. f1:**
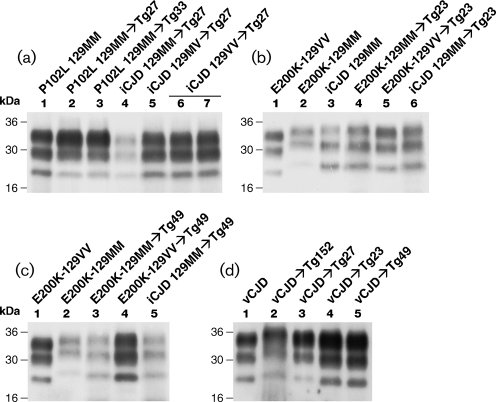
Immunoblot analysis highlighting the PrP^Sc^ types propagated in brains of transgenic mice. Mice were inoculated with classical CJD and prions from patients with IPD P102L and E200K. (a) Immunoblots comparing transmission of IPD P102L prions and classical CJD to 102LL 129M Tg27 and Tg33 transgenic mice. (b) Immunoblots comparing transmission of human isolates from E200K-129MM, E200K-129VV and classical CJD to 200KK 129M Tg23 transgenic mice. (c) Immunoblots comparing transmission of IPD E200K-129MM, P200K-129VV and classical CJD prions to 200KK 129M Tg49 transgenic mice. (d) Immunoblots comparing transmission of vCJD prions to transgenic lines expressing human PrP. The provenance of each brain sample is designated above each lane. Immunoblots were analysed by enhanced chemiluminescence with anti-PrP antibody 3F4.

Within the framework of the protein-only hypothesis, the different phenotypes associated with prion strains are thought to be determined by the propagation of distinct PrP^Sc^ isoforms with divergent physico-chemical properties (Bessen & Marsh, 1994; Collinge *et al.*, 1996b; Telling *et al.*, 1996b; Safar *et al.*, 1998; Prusiner, 1998; Hill & Collinge, 2000; Collinge, 2001; Gambetti *et al.*, 2003; Collinge & Clarke, 2007). IPDs with P102L and E200K mutations are associated with a unique PrP^Sc^ glycoform ratio that differs significantly from those seen in classical CJD and vCJD (Collinge *et al.*, 1996b; Hill *et al.*, 2003, 2006; Wadsworth *et al.*, 2006). We therefore analysed PrP^Sc^ glycoform ratios propagated in human PrP 102L- and 200K-expressing transgenic mice challenged with a range of human prions. Mice inoculated with either IPD isolates or classical CJD isolates propagated PrP^Sc^ with a predominance of both di- and monoglycosylated PrP (Fig. 1). This finding is of particular interest in recipients of classical CJD prions where a change in PrP^Sc^ glycoform ratio is apparent on transmission (see Fig. 1b[Fig f1] lanes 3 and 6; and Table 6). While the primary transmission data suggest that there are statistically significant differences in the glycoform ratios of PrP^Sc^ propagated in human PrP 102L- and 200K-expressing transgenic mice inoculated with IPD isolates (data not shown) or classical CJD prions (Table 6), further subpassage to transgenic mice expressing either mutant or wild-type PrP will be required to fully interpret this observation because of the heterogeneous nature of the primary IPD inocula from patients' brains used in these primary transmissions (Hill *et al.*, 2006; Wadsworth *et al.*, 2006; Wadsworth & Collinge, 2007).

### *PRNP* point mutations in association with 129M do not prevent propagation of the vCJD prion strain

To date all neuropathologically confirmed vCJD cases have been associated with 129M homozygosity (Collinge *et al.*, 1996a; Collinge, 2005). We and others have reported previously that PrP 129M homozygosity is required for the experimental recapitulation of abundant florid plaque formation from BSE and vCJD prion infection (Crozet *et al.*, 2001; Asante *et al.*, 2002; Wadsworth *et al.*, 2004; Bishop *et al.*, 2006). Notably, overexpression is not required for florid plaque formation (Asante *et al.*, 2006). Furthermore, 129V has a dominant-negative effect on the formation of florid plaques in the *PRNP* 129MV heterozygous genotype, leading to dissociation between the propagation of type 4 PrP^Sc^ and florid plaque formation (Asante *et al.*, 2006). To investigate if *PRNP* point mutations would affect the propagation of vCJD prions and expression of characteristic pathology, we challenged Tg27, Tg23 and Tg49 transgenic mice (all of which are homozygous for 129M) with a previously characterized vCJD isolate designated I336 (Tables 2, 4 and 5).

**Fig. 2. f2:**
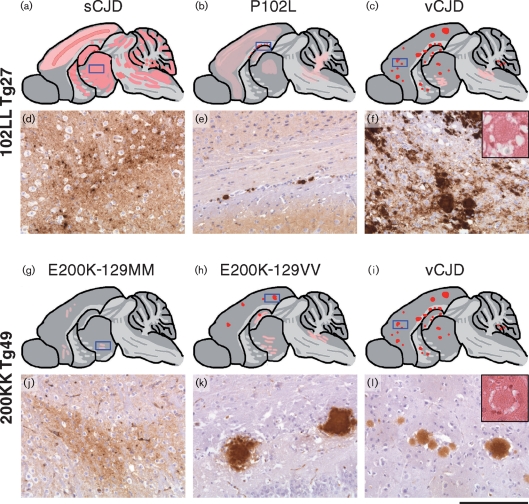
Neuropathological analysis of transgenic mouse brain by PrP immunohistochemistry. Anti-PrP monoclonal antibody ICSM 18 was used for the 102LL transgenic brains, and ICSM 35 was used for the 200KK transgenic brains because ICSM 35 does not detect mutant 102L PrP (Wadsworth *et al.*, 2006). Upper panels (a–c and g–i) show regional distributions of PrP plaque deposition in 102LL 129M Tg27 and 200KK 129M Tg49 transgenic mice challenged with the various human prion inocula with the aetiologies indicated above each panel; synaptic type PrP deposits (pink), discrete PrP plaque deposits (red), blue box in the sketch denotes the area from which the PrP stained sections are derived. Panels (d), (e) and (f) represent PrP monoclonal antibody ICSM 18 immunohistochemical staining in 102LL 129M Tg27 transgenic mice challenged, respectively, with sporadic CJD, P102L and vCJD prions. Panels (j), (k) and (l) represent PrP monoclonal antibody ICSM 35 immunohistochemical staining in 200KK M129 Tg49 transgenic mice challenged, respectively, with IPD E200K-129MM, E200K-129VV and vCJD inocula. Inserts in panels (f) and (l) show examples of florid plaques associated with the neuropathology of vCJD prion transmissions in these mutant mice. Bar, panels (d)–(f) and (j)–(l)=200 μm.

Transmission of vCJD prions to Tg27 mice resulted in only 2/11 clinical attack rate with prolonged incubation periods greater than 482 days post-inoculation (Table 2), but 11/11 total infection rate (positive either by clinical scoring, immunoblot or immunohistochemistry), mirroring transmission properties of the same vCJD inoculum in homozygous human PrP 129MM-expressing Tg35 and Tg45 mice (Asante *et al.*, 2002). Similarly, transmission of vCJD to Tg23 and Tg49 transgenic mice resulted in 3/6 and 2/6 clinical attack rates, respectively, and with prolonged incubation periods (Tables 4 and 5), and these were again accompanied by 100 % total infection rate in both lines, as determined either by clinical scoring, Western blot analysis or immunohistochemistry. Affected Tg27 mice propagated PrP^Sc^ that was closely similar to type 4 PrP^Sc^ present in the vCJD inoculum (Fig. 1d[Fig f1], lanes 1 and 3) and distinct from type 5 PrP^Sc^ seen in vCJD-inoculated Tg152 mice expressing human PrP 129V (Hill *et al.*, 1997; Wadsworth *et al.*, 2004) (Fig. 1d[Fig f1], lane 2). In keeping with this finding, vCJD-inoculated Tg27 mice showed neuropathological changes that were characteristic of the vCJD prion strain with extensive plaque deposition many of which were of the florid type (Fig. 2c, f[Fig f2]).

In vCJD challenged human PrP 200K-expressing transgenic mice Tg23 (data not shown) and Tg49 (Fig. 2i, l[Fig f2]), abundant florid plaques were also observed that were indistinguishable from the florid plaques generated in transgenic mice expressing wild-type human PrP 129MM, Tg35 or Tg45 (Asante *et al.*, 2002). Interestingly, the lower human PrP 200K-129M-expressing Tg49 line had a higher PrP plaque load than Tg23 mice, suggesting that PrP plaque density may be related to the kinetics of PrP^Sc^ formation. Notably, however, both human PrP 200K-129M-expressing lines Tg23 and Tg49 propagated PrP^Sc^ with a slightly lower molecular mass fragment size than type 4 PrP^Sc^ seen in the vCJD inoculum (Fig. 1d[Fig f1], lanes 4 and 5). We have provisionally designated this new PrP^Sc^ isoform, that generates proteinase K-resistant fragments sharing the glycoform ratio of types 4 and 5 PrP^Sc^ but with a smaller fragment size than type 4, as human PrP^Sc^ type 8. Serial passage studies will be required to establish if, like type 5 PrP^Sc^, this novel PrP^Sc^ type represents a distinct vCJD-derived prion strain. In this regard, it will be interesting to see whether this distinct PrP^Sc^ conformer, that is associated with abundant florid plaques in human PrP 200K-expressing transgenic mice (Fig. 2i, l[Fig f2]), can be maintained on subpassage in transgenic mice expressing wild-type human PrP.

## DISCUSSION

In these studies, we have used transgenic mice homozygous for two different human PrP mutations and devoid of murine PrP, in order to allow a comparative study of the transmission properties of *PRNP* mutations in the absence of the confounding effects of endogenous murine PrP. Our study differs from previous reports (Hsiao *et al.*, 1990; Hegde *et al.*, 1998; Chiesa *et al.*, 1998; Manson *et al.*, 1999), in that we have modelled *PRNP* disease-associated mutations on human PrP, rather than superimposing the human mutations on rodent PrP. This is particularly important because destabilizing effects measured in a mouse protein cannot be assumed to be equivalent in the human protein (Wildegger *et al.*, 1999; T. Hart, G. J. Jackson, A. R. Clarke & J. Collinge, unpublished data).

Our data show that while the P102L mutation is permissive to homotypic IPD P102L prions, there appears to be a barrier limiting the transmission of classical CJD prions. In contrast, the E200K mutation is equally permissive to homotypic IPD E200K and classical CJD prions, providing there is no mismatch at *PRNP* codon 129. Neither mutation appears to influence the propagation of the vCJD prion strain.

Previously we have shown that cases of IPD caused by the *PRNP* point mutations P102L, D178N and E200K have a unique PrP^Sc^ glycoform ratio following proteinase K digestion, which differs significantly from PrP^Sc^ glycoform ratios observed in sporadic, iatrogenic and vCJD or IPD caused by octapeptide repeat insertional mutations (Hill *et al.*, 2006; Wadsworth *et al.*, 2006). These data suggested that point mutations in *PRNP* either destabilize non-glycosylated PrP, in turn reducing its relative abundance (Petersen *et al.*, 1996), or directly dictate the stoichiometry and packing order of the three PrP glycoforms into disease-related fibrils or other aggregates. The latter explanation is consistent with a conformational selection model of prion transmission barriers (Collinge, 1999; Hill & Collinge, 2003; Collinge & Clarke, 2007) that predicts that coding changes in PrP act to specify structural preferences for disease-related PrP isoforms. The full spectrum of effects that different pathogenic *PRNP* mutations may have still remains unclear (Hill *et al.*, 2006).

Importantly, our data now show that the PrP^Sc^ glycoform ratio of classical CJD prions is not maintained on passage in transgenic mice expressing *PRNP* 102L or 200K point mutations. Instead PrP^Sc^ propagates with a glycoform ratio closely similar to those seen in patients with P102L and E200K IPD that is significantly different from the PrP^Sc^ types present in the classical CJD inoculum. These data support the hypothesis that prion strains propagated in IPDs are distinct from those propagated in classical (sporadic and iatrogenic) CJD (Hill *et al.*, 2006). As equivalent proportions of the PrP glycoforms are seen in PrP^C^ expressed in uninoculated wild-type or mutant *PRNP* transgenic mice, this change in PrP^Sc^ glycoform ratio is consistent with the *PRNP* point mutations acting to directly dictate the stoichiometry and packing order of the three PrP glycoforms into disease-related fibrils or other aggregates (Hill *et al.*, 2006). Furthermore, previous immunoprecipitation studies using a panel of monoclonal antibodies suggested that the proportion of each glycoform incorporated into PrP^Sc^ is probably controlled in a strain-specific manner (Khalili-Shirazi *et al.*, 2005).

The interpretation of primary transmission data for IPD isolates is complicated by the heterogeneous composition of disease-related PrP isoforms that may be present in the primary inoculum (Hill *et al.*, 2006; Wadsworth *et al.*, 2006; Wadsworth & Collinge, 2007). For example in P102L IPD, it is now apparent that three isoforms of protease-resistant PrP with divergent physico-chemical properties can be propagated. Two distinct abnormal conformers derived from PrP P102L generate protease-resistant fragments of either approximately 21–30 or 8 kDa (Parchi *et al.*, 1998; Piccardo *et al.*, 1998, 2007; Muramoto *et al.*, 2000; Hill *et al.*, 2006; Wadsworth *et al.*, 2006), while abnormal conformers of wild-type PrP appear to generate proteolytic fragments of only approximately 21–30 kDa (Wadsworth *et al.*, 2006). Glycoform ratios of approximately 21–30 kDa proteolytic fragments generated from PrP P102L and wild-type PrP are not only distinct from each other, but are also distinct from those generated from wild-type PrP in sporadic or acquired CJD (Wadsworth *et al.*, 2006). Differences in neuropathological targeting of these distinct disease-related PrP species, together with differences in their abundance and potential neurotoxicity, provide a molecular mechanism for generation of multiple phenotypes in P102L IPD (Wadsworth *et al.*, 2006; Piccardo *et al.*, 2007). Propagation of particular PrP^Sc^ isoforms in a new host will also be determined by host genetic background, *PRNP* sequence and prion strain type (Collinge & Clarke, 2007). These data have significant implications for interpreting the transmission properties of IPD isolates in both conventional and transgenic mice and may in part explain the historical differences seen in previous transmissions of IPD isolates (Brown *et al.*, 1994; Tateishi *et al.*, 1996). Serial passage studies of the prion isolates generated here should help to clarify the major influencing factors limiting the transmission of IPD, and how many strains may be associated with IPDs.

## Figures and Tables

**Table 1. t1:** Characterization of mutant human PrP transgenic lines For each line, a cohort of mice was set aside for long-term observation. 102LL homozygous for proline to leucine 102 mutation; 200KK homozygous for glutamic acid to lysine 200 mutation; nd, not determined.

**PrP sequence**	**Tg-line**	Transgene copy number*	**Expression level†**	**Age of oldest mouse (days)**	**Mean survival (days±sem)**	**Development of spontaneous neuropathology‡**
102LL, 129MM	Tg27	11	3	870	736±36 (*n*=9)	No
	Tg33	5	1.5	870	679±43 (*n*=13)	No
200KK, 129MM	Tg23	5	3	960	832±34 (*n*=14)	No
	Tg49	nd	2	862	753±60 (*n*=5)	No

*Transgene copy number was determined in hemizygous mice. †Expression levels presented here are homozygous levels and are relative to pooled normal human brain homogenate levels. ‡Each brain was scored negative for disease-related PrP by immunohistochemistry and immunoblotting.

**Table 2. t2:** Classical CJD and IPD P102L prion transmissions to human PrP 102LL 129M Tg27 transgenic mice IHC, Immunohistochemistry; IB, immunoblotting; DM, dura mater; GH, growth hormone; nd, not determined.

**Aetiology**	**Inoculum**			**Tg(HuPrP^102L,129M+/+^*Prnp*^o/o^)-27**				
	**Code**	***PRNP* 129 genotype**	Human PrP^Sc^ type*	**Clinical signs**	**Incubation period (days±sem)**	**Positive by IHC†**	**Positive by IB†**	**Total affected‡**
IPD P102L	I1087	MV§	T1	8/8	191±0.4	nd	8/8||	8/8
	I1479	MM	T1	9/9	182±3	2/2	8/8||	8/8
	I1480	MM	T1	7/9	181±3	nd	9/9||	9/9
	I1476	MV§	T1	11/12	187±7	nd	12/12||	12/12
Sporadic CJD	I1200	MM	T1	5/10	717±22	7/7	9/9||	10/10
Iatrogenic CJD	I026 (DM)	MM	T2	3/8	653±83	5/6	5/6||	6/8
	I020 (GH)	MV	T3	6/7	411±7	2/2	6/6	7/7
	I021 (GH)	VV	T3	8/10	342±20	5/5	7/7||	10/10
vCJD	I336	MM	T4	2/11	483, 498	9/9	10/11¶	11/11

*According to the classification of Hill *et al.* (2003). †Primary antibody was monoclonal ICSM 18 because monoclonal antibody ICSM 35 does not recognize 102L PrP^Sc^ (Wadsworth *et al.*, 2006). ‡Positive either by clinical signs, Western blot analysis and/or immunohistochemistry. §*PRNP* mutation is on the 129M allele (Hill *et al.*, 2006). ||Glycoform profile for all samples showed a shift towards diglycosylated dominance (Hill *et al.*, 2006). ¶All positive samples propagated T4 PrP^Sc^ (Hill *et al.*, 2003).

**Table 3. t3:** Classical CJD and IPD P102L prion transmissions to human PrP 102LL 129M Tg33 transgenic mice IHC, Immunohistochemistry; IB, immunoblotting; DM, dura mater.

**Aetiology**	**Inoculum**			**Tg(HuPrP^102L,129M+/+^*Prnp*^o/o^)-33**				
	**Code**	***PRNP* 129 genotype**	Human PrP^Sc^ type*	**Clinical signs**	**Incubation period (days±sem)**	**Positive by IHC†**	**Positive by IB†**	**Total affected‡**
IPD P102L	I1087	MV§	T1	1/8	355	3/3	8/8||	8/8
	I1088	MM	T2	4/8	297±4	6/6	6/6||	8/8
	I1090	MM	T2	4/8	304±13	3/3	8/8¶	8/8
Sporadic CJD	I282	MM	T1	2/3	510, 515	2/3	2/3#	2/3
	I284	MV	T2	1/5	547	3/4	0/5**	3/5
Iatrogenic CJD (DM)	I020	MV	T3	0/3	>445	2/2	3/3††	3/3

*According to the classification of Hill *et al.* (2003). †Primary antibody was monoclonal ICSM 18 because monoclonal antibody ICSM 35 does not recognize P102L prions (Wadsworth *et al.*, 2006). ‡Positive either by clinical signs, Western blot analysis and/or immunohistochemistry. §*PRNP* mutation is on the 129M allele (Hill *et al.*, 2006). ||Glycoform profile for all samples was the inherited PrP^Sc^ pattern (Hill *et al.*, 2006). ¶Glycoform profile for all samples showed a shift towards diglycosylated dominance (Hill *et al.*, 2006). #Two samples had the inherited PrP^Sc^ glycotype and differed significantly from the classical CJD type (Hill *et al.*, 2006). **No sample was positive by IB. ††Signal was too weak to allow assignment of glycotypes.

**Table 4. t4:** Classical CJD and IPD E200K prion transmissions to human PrP 200KK 129M Tg23 transgenic mice IHC, Immunohistochemistry; IB, immunoblotting; DM, dura mater.

**Aetiology**	**Inoculum**			**Tg(HuPrP^200K,129M+/+^*Prnp*^o/o^)-23**				
	**Code**	***PRNP* 129 genotype**	Human PrP^Sc^ type*	**Clinical signs**	**Incubation period (days±sem)**	**Positive by IHC†**	**Positive by IB†**	**Total affected‡**
Iatrogenic CJD (DM)	I026	129MM	T2	8/8	184±7	2/2	5/5§	8/8
IPD E200K	I1091	129MM	T1	8/8	184±3	2/2	4/4||	8/8
IPD E200K	I1093	129VV	T3	1/4	437	3/3	4/4¶	4/4
vCJD	I336	129MM	T4	3/6	578	3/3	5/5#	6/6

*According to the classification of Hill *et al.* (2003). †Primary antibody was monoclonal ICSM 35. ‡Positive either by clinical signs, Western blot analysis and/or immunohistochemistry, primary antibody was ICSM 35. §Four samples propagated inherited PrP^Sc^ pattern (Hill *et al.*, 2006), one sample was positive but signal was too weak to assign glycotype. ||Three samples propagated inherited PrP^Sc^ pattern (Hill *et al.*, 2006), one sample was positive but signal was too weak to assign glycotype. ¶All positive samples propagated the inherited PrP^Sc^ pattern (Hill *et al.*, 2006). #All positive samples propagated a novel human PrP^Sc^ type (designated PrP^Sc^ type 8) with lower molecular mass fragment size than type 4 PrP^Sc^ (Hill *et al.*, 2003).

**Table 5. t5:** Classical CJD and IPD E200K prion transmissions to human PrP 200KK 129M Tg49 transgenic mice IHC, Immunohistochemistry; IB, immunoblotting; DM, dura mater.

**Aetiology**	**Inoculum**			**Tg(HuPrP^200K,129M+/+^*Prnp*^o/o^)-49**				
	**Code**	***PRNP* 129 genotype**	**Human PrP^Sc^ type**	**Clinical signs**	**Incubation period (days±sem)**	Positive by IHC*	Positive by IB*	**Total affected†**
Iatrogenic CJD (DM)	I026	129MM	T2	6/9	344±8	4/5	8/8‡	9/9
IPD E200K	I1091	129MM	T1	5/8	348±22	5/6	8/8‡	8/8
IPD E200K	I1093	129VV	T3	1/6	552	4/5	6/6‡	6/6
vCJD	I336	129MM	T4	2/6	631, 681	4/4	5/5§	6/6

*Primary antibody was monoclonal ICSM 35. †Positive either by clinical signs, Western blot analysis and/or immunohistochemistry; primary antibody was ICSM 35. ‡All positive samples propagated the inherited PrP^Sc^ pattern (Hill *et al.*, 2006). §All positive samples propagated a novel PrP^Sc^ type (designated PrP^Sc^ type 8) with lower molecular mass fragment size than type 4 PrP^Sc^ (Hill *et al.*, 2003).

**Table 6. t6:** Transmission of classical CJD leads to a significant alteration in PrP^Sc^ glycoform ratios in 200KK and 102LL transgenic mice

***PRNP* mutation**	**Classical CJD T2 MM**	**200KK 129M Tg23**	**200KK 129M Tg49**	**102LL 129M Tg27**
	None	E200K	E200K	P102L
Diglycosylated PrP^Sc^	19.6±1.0*	32.6±2.0 *P*<0.0001	40.9±4.0 *P*<0.0001	50.8±5.0 *P*<0.0001
Monoglycosylated PrP^Sc^	47.9±1.0	48.3±2.0 *P*=0.8	37.0±2.0 *P*<0.0001	36.5±2.0 *P*<0.0001
Unglycosylated PrP^Sc^	32.6±1.0	19.1±1.0 *P*<0.0001	21.8±2.0 *P*<0.0004	12.6±3.0 *P*<0.0001

*Glycoform ratios of PrP^Sc^ propagated in transgenic mice (*n*=3 per line) inoculated with classical CJD isolate I026 [type 2 PrP^Sc^ 129MM (T2 MM)] are compared with the human T2 MM glycoform ratio (*n*=11) (Hill *et al.*, 2006) Data show mean±sem. *P* values relate to comparison with classical CJD T2 MM (unpaired two-tailed *t*-test).
